# Combination of Botulinum Toxin A and Hyaluronic Acid Improved Facial Pore Enlargement Caused by Acne

**DOI:** 10.1111/jocd.70198

**Published:** 2025-04-24

**Authors:** Rongli Yang, Yiyang Bai, Chang Liu, Jintian Luo, Yajie Yang, Shaoli Cheng, Muyan Zou, Xin Mu

**Affiliations:** ^1^ Department of Dermatology The First Affiliated Hospital of Xi'an Jiaotong University Xi'an Shaanxi China; ^2^ Department of Dermatology Weinan Central Hospital Weinan Shaanxi China; ^3^ Zhongshan School of Medicine, Sun Yat‐Sen University Guangzhou Guangdong China; ^4^ Basic Medical Experiment Teaching Center Health Science Center, Xi'an Jiaotong University Xi'an Shaanxi China; ^5^ Medical Department Imeik Technology Development Co., Ltd. Beijing China

**Keywords:** botulinum toxin A, enlarged pores, hyaluronic acid, therapeutic effect

## Abstract

**Background:**

Acne often leads to enlarged pores on the facial skin, seriously affecting the patient's appearance. Topical and oral medications have poor efficacy in some cases of severe pore enlargement.

**Objective:**

This study aimed to explore the clinical efficacy and safety of botulinum toxin A combined with hyaluronic acid in improving facial pore enlargement.

**Method:**

A retrospective study was conducted on 40 acne patients with enlarged pores. Botulinum toxin A and non‐cross‐linked hyaluronic acid compound were injected into the patient's face using mesotherapy with nine 32‐gauge microneedles. VISIA was used to record the conditions of facial pores, texture, and porphyrins. The physician's assessment of the improvement score and the patient satisfaction evaluation are used to evaluate the treatment effect.

**Result:**

One month after treatment, the combination of botulinum toxin and non‐cross‐linked hyaluronic acid compound significantly improved facial pore enlargement. Although the effects decreased, the conditions of facial pores, texture, and porphyrins 4 months after treatment still had a significant difference from those at baseline.

**Conclusion:**

The combination of botulinum toxin A and non‐cross‐linked hyaluronic acid compound is safe and effective in treating enlarged pores, indicating its promotional value.

## 1 Introduction

Acne is a chronic inflammatory skin disease that is still common in adults. It affects the pilosebaceous unit and manifests as comedones, papules, pustules, nodules, cysts, and scars. The pathogenesis of acne is believed to include abnormal keratinization of the sebaceous duct, excessive sebum secretion, inflammation, and proliferation of *Cutibacterium acnes* [1]. The repeated occurrence of acne can lead to enlarged pores and scar formation, seriously affecting the patient's appearance.

The pores of the skin serve as the outlet for the pilosebaceous unit. The sebaceous gland is a type of gland without a cavity. It is composed of glands and ducts and is mostly located between the hair follicle and the erector pili muscle. The ducts mostly open in the upper part of the hair follicle. Some directly open on the surface of the skin. Pores are prominent in the nose and inner cheek skin where the sebaceous glands are concentrated on the face. The sebaceous gland secretion and pore size are positively correlated. An increase in the number of pores and sebum secretion can be observed on the face of acne patients. Excessive secretion of facial sebum is often considered the main cause of acne with enlarged pores [2].

The treatment for enlarged pores mainly involves reducing sebum secretion, improving skin photoaging to restore skin elasticity, and reducing hair follicle size. Antiandrogenic drugs are the most commonly used oral medications that inhibit the secretion of the sebaceous glands [3]. Isotretinoin, the most effective sebum inhibitor, can inhibit sebaceous gland secretion. It is used in treating moderate to severe acne and inflammatory diseases of hair follicles and sebaceous glands accompanied by sebum leakage. However, oral isotretinoin causes many side effects during and after the treatment [4]. Local topical retinoic acid is used to treat skin photoaging and can remove keratin plugs by regulating the abnormal keratinization of hair follicles; as a result, enlarged pores can be improved. However, this acid may cause contact dermatitis [5]. Chemical peeling has also been applied to alleviate pore enlargement. α‐Hydroxylic acid (fruit acid) and β‐hydroxyl acids (salicylic acid) are used in chemical peel, which promotes epidermal cell turnover and initiates dermal repair and reconstruction [6]. The main mechanism by which phototherapy reduces pore enlargement may involve the reconstruction of collagen fibers near the opening of hair follicles under thermal energy; this mechanism increases skin elasticity and reduces sebum secretion, thereby improving pore enlargement [7]. These therapies require long‐term treatment to have a certain effect.

Hyaluronic acid (HA) plays an important physiological role in maintaining water retention, maintaining extracellular space, and regulating osmotic pressure. Intradermal injection of non‐cross‐linked small molecule HA has moisturizing, antiwrinkling, and whitening effects. Facial injection can increase skin moisture, enhance skin elasticity and toughness, promote new collagen production, significantly improve skin texture, and reduce pore size [8].

Intradermal injection of type A botulinum toxin (BTX‐A) can shrink pores, possibly by reducing sebum secretion [9]. Anil found that 85% of patients show improved sebum production and decreased pore size after 1 month of intradermal injection of BTX‐A [10]. However, another study found that the percutaneous injection of BTX‐A significantly improves skin texture but does not significantly reduce pore size and sebum production [11]. The results of the above research suggest that the simple injection of BTX‐A still cannot change the pore size of some patients.

BTX‐A reduces pore size by inhibiting sebum production, whereas HA can improve skin texture. The combination of BTX‐A and HA can perfectly reduce pore size and improve skin texture, thereby achieving good aesthetic effects. The combination therapy of BTX‐A and HA fillers can address wrinkles and the resulting volume loss from the deep tissue layers. This combination method is becoming increasingly popular [12, 13]. The Global Aesthetic Consensus Group (GACG) has recently recognized the value of combination therapy. Continuous combination therapy may result in cumulative improvement when volume reduction leads to the presence of deep wrinkles. GACG conducted a survey on the practice mode, and the results indicated that 21% of patients used combination therapy in the eyebrow area [12]. However, little evidence supports the efficacy of combination therapy. Moreover, combining the two for enlarged pore treatment is rarely reported. Therefore, this study used BTX‐A combined with non‐cross‐linked HA compound to treat facial skin. The clinical efficacy and safety of this combination in treating enlarged pores were also observed.

## 2 Materials and Methods

### 2.1 Patients

Forty patients with acne with enlarged pores, aged 25 ± 6, were selected from the outpatient department of the First Affiliated Hospital of Xi'an Jiaotong University's dermatology department. Among them, 32 were females and 8 were males. According to Fitzpatrick skin classification, 12 patients were classified as type III, and 28 were classified as type IV. All patients were informed of the entire treatment process and signed informed consent forms.

The inclusion criteria are as follows: (1) age range from 18 years to 40 years, (2) acne with enlarged pores diagnosed by two experienced dermatologists, (3) voluntary participation in this trial and a signed informed consent form, and (4) completed follow‐up appointments.

The exclusion criteria are as follows: (1) women preparing for pregnancy, pregnant women, or lactating women; (2) local medication within 3 months or systemic medication within 6 months for enlarged pore treatment; (3) chemical rejuvenation, facial cosmetic surgery, phototherapy, or HA or BTX‐A treatment within 6 months; (4) facial complications of other inflammatory or tumor‐related diseases; (5) poor wound healing and keloids; (6) allergy to any component of HA or BTX‐A.

### 2.2 Method

**FIGURE 1:**
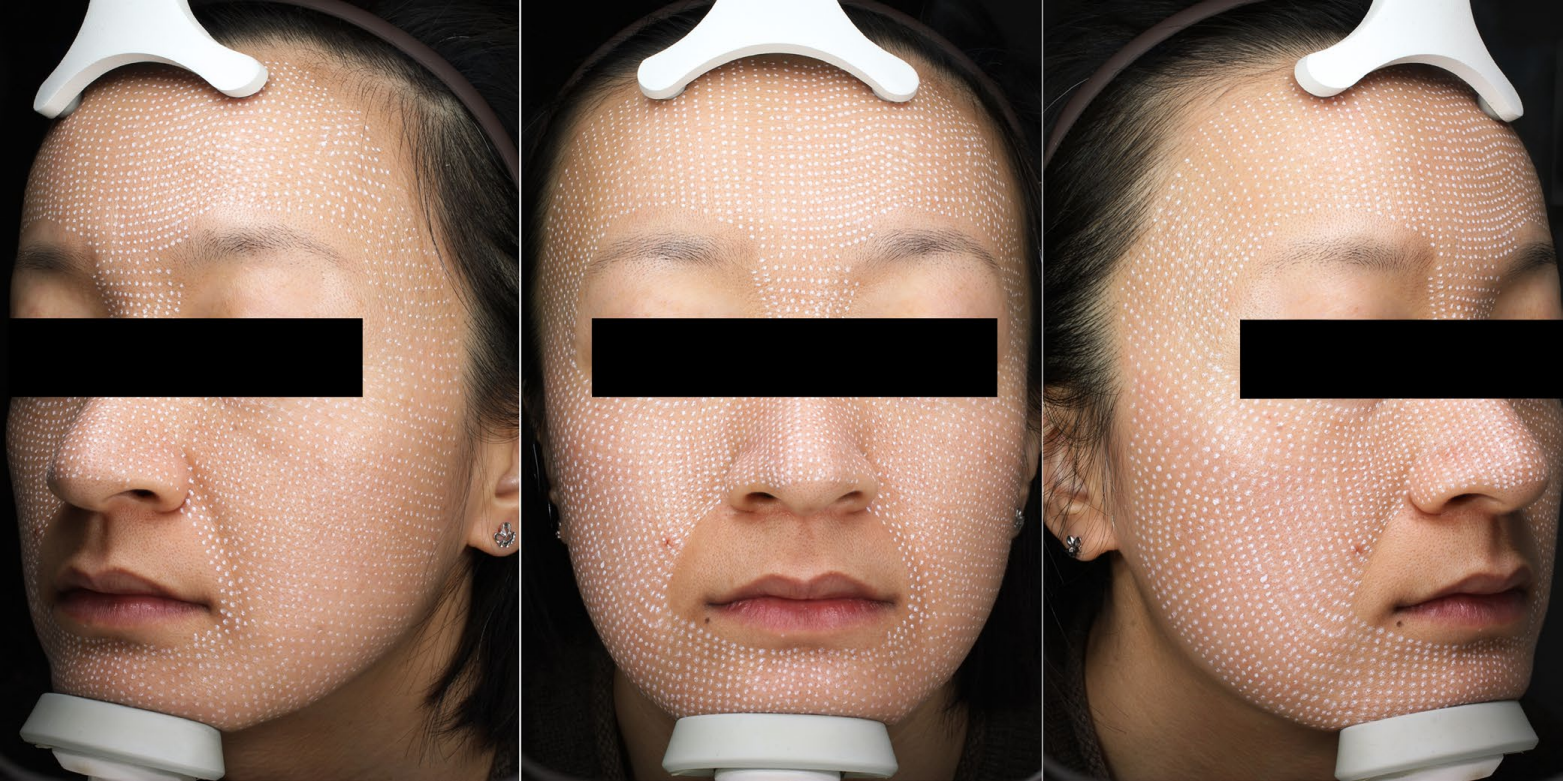
Represent diagram of the injection point of BTX‐A and HA on face.

The patient's face was cleaned before the injection process. The full‐face images were captured by VISIA. Then, 100 U of BTX‐A (Hengli, Lanzhou Institute of Biological Products, Lanzhou City, China) was dissolved in 2.5 mL of sterile saline (40 U/mL of BTX‐A solution). Afterward, 30–50 U of BTX‐A was added to 2.5 mL of non‐cross‐linked HA compound (sodium hyaluronate, 5.00 mg/mL; l‐carnosine, 2.00 mg/mL; profile, 0.20 mg/mL; glycine, 0.10 mg/mL; alanine, 0.10 mg/mL; vitamin B2, 0.005 mg/mL) (Heart, Imeik Inc. Ltd., Beijing, China). The automatic intradermal injector, Derma Shine Mesotherapy (Huons, Seongnam, Korea), was used for this treatment. The automatic injector was combined with a multineedle applicator comprising nine 32‐gauge microneedles. An appropriate amount of lidocaine cream (10 g/tube, Tongfang Pharmaceutical Group Co. Ltd., Beijing, China) was applied to the entire face. After 40 min, the lidocaine cream was removed, and the skin was disinfected. The skin was treated using circulating negative pressure suction injection. The multineedle applicator was inserted into specific skin layers. After injection, the negative pressure disappeared, and the syringe and skin automatically separated. The injection volume at each point was 0.0278 mL to 0.0333 mL of mixed solution. The injection depth was approximately 0.8–1 mm. The injection points of BTX‐A and HA in the face are demonstrated as shown in Figure 1.

### 2.3 Objective Indicators

The VISIA Skin Image Analyzer (Canfield Imaging Systems, Fairfield, NJ, USA) was used to record the condition of facial pores, texture, and porphyrins at baseline, 1 month after treatment, and 4 months after treatment. The patients' facial photographs (front, left 45°, and right 45°) were obtained using VISIA at baseline, 1 month after treatment, and 4 months after treatment. The patients were advised not to use cosmetics and skin care products on the day the photo would be taken. On the day the photo was taken, their faces were cleaned with running water; after 30 min, the photo was taken. The pores, texture, and porphyrins were evaluated and compared. A low score represents small pores, fine and smooth skin, and a decrease in sebum secretion.

### 2.4 Physician Assessment

On the basis of the optical photographs and the VISIA images of patients at baseline, 1 month after treatment, and 4 months after treatment, two dermatologists who did not participate in the treatment scored the changes in skin pores, texture, and porphyrins using the Global Aesthetic Improvement Scale (GAIS). GAIS was used by dermatologists to grade changes in skin pores, texture, and porphyrins. The score range based on the degree of improvement is from −1 point to 3 points: −1 (worse), 0 (no change), 1 (improved), 2 (much improved), and 3 (very much improved). A GAIS score of 1 or above indicates that the treatment is effective.

### 2.5 Overall Patient Satisfaction Assessment

Patient satisfaction was evaluated during follow‐up appointments 1 month and 4 months after treatment. The overall subjective satisfaction [14] is divided into five levels, and the corresponding scores are 0 (worse), 1 (poor satisfaction), 2 (fairly satisfied), 3 (satisfied), and 4 (very satisfied). The higher the score is, the higher the satisfaction is.

### 2.6 Safety Evaluation

The same physician asked the patients if they experienced symptoms such as pain, burning sensation, redness, swelling, and allergic reactions during the injection therapy. The physician objectively evaluated the postoperative skin bruising, facial expression stiffness, asymmetry, and skin tightness. The severity was recorded. The patients were asked if any discomforts, such as bruising, stiff facial expressions, asymmetry, muscle weakness, difficulty swallowing, dry mouth, fatigue, headache, blepharoptosis, and difficulty pronouncing, were experienced during the follow‐up periods of 1 month and 4 months. All adverse reactions were recorded.

### 2.7 Statistical Analysis

SPSS 18.0 software was used for statistical data analysis. The Kruskal–Wallis test was used to detect facial pores, texture, and porphyrins. Moreover, *p* < 0.05 indicates a statistically significant difference.

## 3 Results

### 3.1 VISIA Values of Pores, Texture, and Porphyrins

**FIGURE 2:**
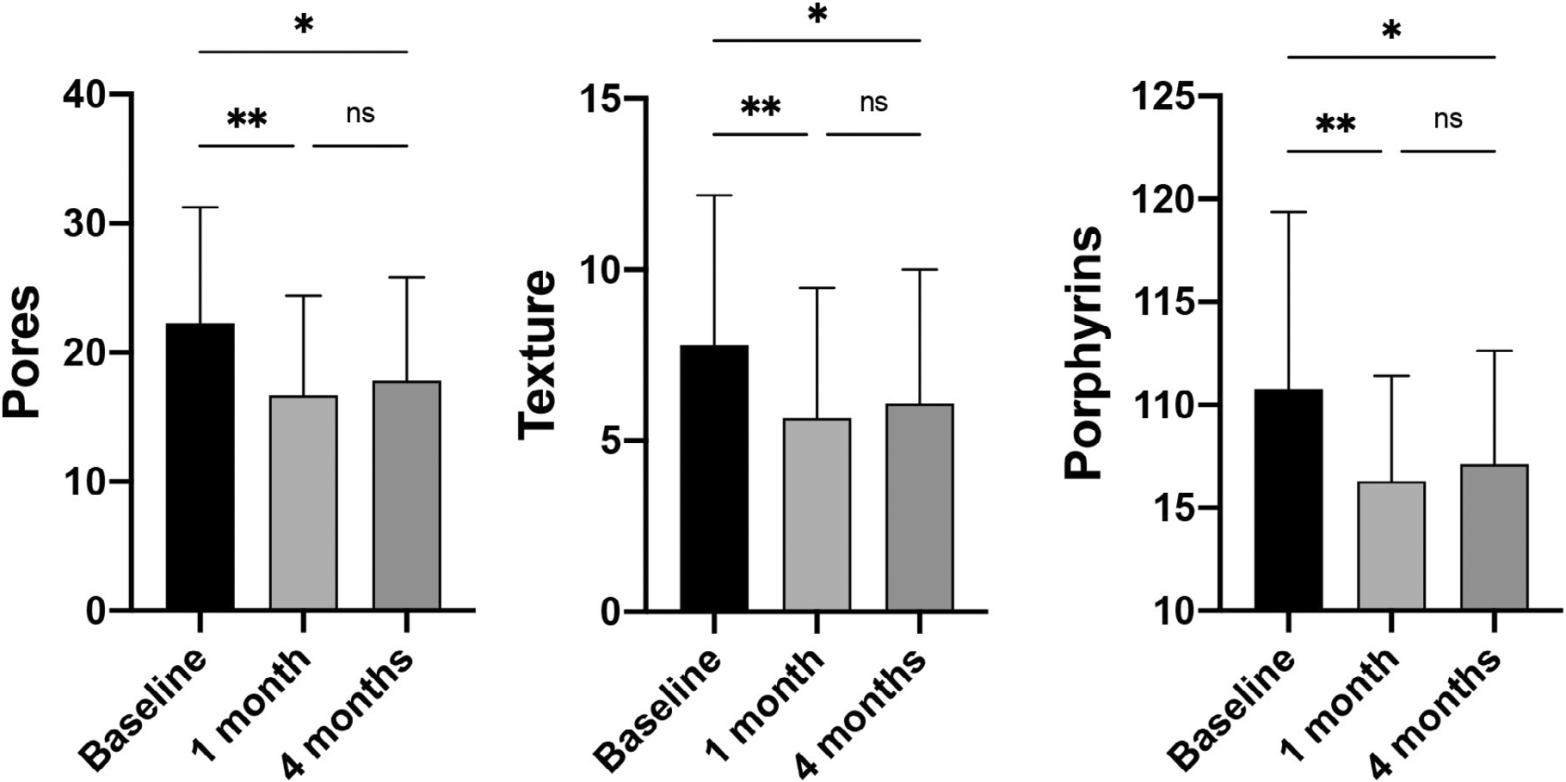
The pores, texture, and porphyrins assessed by VISIA at baseline, 1 month after treatment, and 4 months after treatment (**p < 0.01; *p < 0.05; ^ns^ *p* > 0.05).

VISIA detection results indicate that the pore value at baseline, 1 month after treatment, and 4 months after treatment was 22.3 ± 9.0, 16.7 ± 7.7, and 17.8 ± 8.0, respectively. The pore value at baseline and that 1 month after treatment had a statistically significant difference (*p* < 0.01); the pore values at baseline and 4 months after treatment had a statistically significant difference (*p* < 0.05); the pore values 1 month and 4 months after treatment had no statistically significant difference (*p* > 0.05). The texture value at baseline, 1 month after treatment, and 4 months after treatment was 7.8 ± 4.4, 5.7 ± 3.9, and 6.1 ± 4.0, respectively. The texture values at baseline and 1 month after treatment had a statistically significant difference (*p* < 0.01); the texture values at baseline and 4 months after treatment had a statistically significant difference (*p* < 0.05); the texture values 1 month and 4 months after treatment had no statistically significant difference (*p* > 0.05). The porphyrin value at baseline, 1 month after treatment, and 4 months after treatment was 10.8 ± 8.6, 6.3 ± 5.1, and 7.1 ± 5.5, respectively. The porphyrin values at baseline and 1 month after treatment had a statistically significant difference (*p* < 0.01); the porphyrin values at baseline and 4 months after treatment had a statistically significant difference (*p* < 0.05); the porphyrin values 1 month and 4 months after treatment had no statistically significant difference (*p* > 0.05) (Figure 2).

**FIGURE 3:**
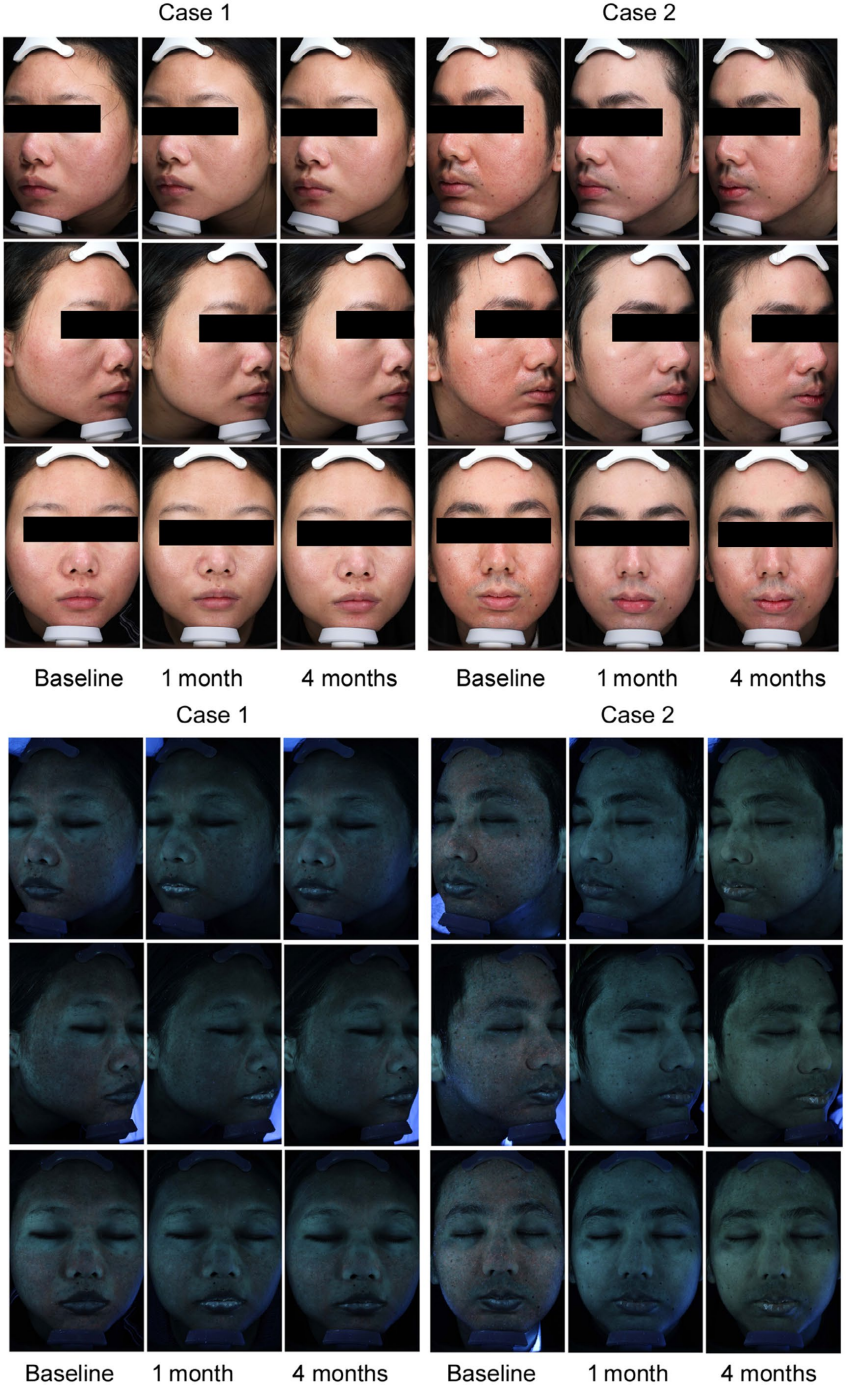
Facial responses of typical patients (a 25‐year‐old female and a 26‐year‐old male patient) at baseline, 1 month after treatment, and 4 months after treatment. Noticeable improvement can be observed 1 month after treatment. Moreover, the improvement degree 1 month after treatment is better than that 4 months after treatment.

The VISIA results of pores, texture, and porphyrins indicated that the patient's pores were significantly improved after the BTX‐A and HA combination treatment. Porphyrins were decreased, indicating a decrease in sebum secretion. Furthermore, Figure 3 presents a graphical illustration of the VISIA data for facial pore patients. The images depict the status of pores and porphyrins at baseline and 1 month and 4 months following the final treatment session.

### 3.2 Improvement Assessment by the Physician

**FIGURE 4:**
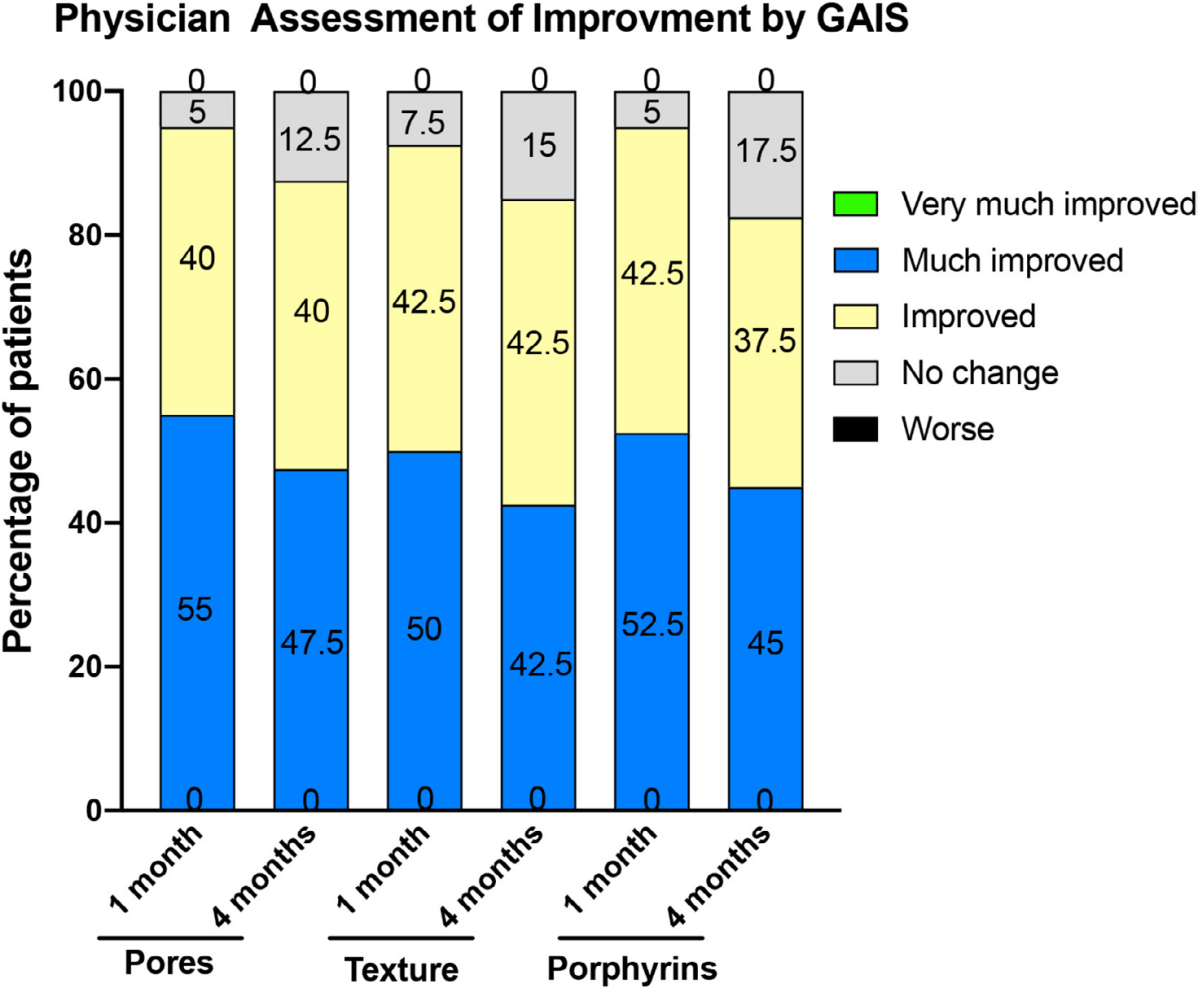
Evaluator‐rated change in patient's facial pores, texture, and porphyrins according to the GAIS score 1 month and 4 months after treatment. Bars represent the percentage of patients in each category assessed.

One month after treatment, the physician's GAIS evaluation showed that the patient's facial pores were improved to varying degrees, with an effectiveness rate of 95% (including 40% improved and 55% much improved) in pores. Among the 40 patients, only 5% (2 patients) had no changes in pores after treatment. No patients experienced worsening in terms of the pore value after treatment. The improvement in texture and porphyrins was 92.5% (including 42.5% improved and 50% much improved) and 95% (including 42.5% improved and 52.5% much improved), respectively. This finding indicates that the sebum secretion after treatment decreased in 38 patients compared with that at baseline. The GAIS score 4 months after treatment showed that the effectiveness rate for improving pores, texture, and porphyrins was 87.5% (including 40% improved and 47.5% much improved), 85% (including 42.5% improved and 42.5% much improved), and 82.5% (including 37.5% improved and 45% much improved), respectively. The effectiveness rates for improving pores, texture, and porphyrins 4 months after treatment were lower than those 1 month after treatment (Figure 4).

### 3.3 Satisfaction Evaluation of Patients

The satisfaction rate of patients 1 month and 4 months after treatment was 87.5% and 77.5%, respectively. The satisfaction rate after 4 months of treatment decreased by 10% compared with that after 1 month of treatment (Table 1).

**TABLE 1:** Overall satisfaction of 40 patients at 1 and 4 months after treatment.

Month	Worse	Poor satisfaction	Relatively satisfaction	Satisfaction	Very satisfaction	Overall satisfaction rate (*n*)%
1	2	3	8	18	9	87.5
4	4	5	12	14	5	77.5

### 3.4 Safety Evaluation

All 40 patients experienced mild, tolerable pain during BTX‐A combined with HA injection. The pain was immediately relieved after injection. Erythema in the cheek area occurred in three cases after injection. The erythema decreased after 20 min of cold compress. Five cases indicated bruising in the lower eyelid area after injection, with approximately 2–5 bruising spots. The bruising gradually subsided after 5 days. Two cases reported tightness in the cheek area after 1 week of injection, and the symptoms disappeared 1 month later. All patients did not experience severe adverse reactions, such as facial asymmetry, eyebrow asymmetry, allergic reactions, muscle weakness, respiratory distress, and fatigue. 4 months after treatment, four patients experienced recurrent symptoms of enlarged pores and requested further treatment.

## 4 Discussion

In this study, the combination of BTX‐A and HA was used to improve enlarged pores resulting from acne. The VISIA results for facial pores, texture, and porphyrins, the physician's GAIS evaluation, and the patient satisfaction evaluation suggested that the combined application significantly improved enlarged pores.

BTX‐A can be applied to enlarged pores. A single intradermal microinjection of BTX‐A can effectively control sebum and pore size. Its effects on the wrinkles in the nasolabial fold last 12 weeks [15]. In a study of 18 volunteers, the intradermal injection of BTX‐A has no significant effect on wrinkles and sebum secretion in the infraorbital region. The improvement in the nasal and lip wrinkles lasts for 12 weeks. The improvement in skin texture lasts for 8 weeks, but the improvement in pores is only observed in the 2nd week [16]. A split‐face controlled pilot study showed that BTX‐A treatment significantly reduces sebum and facial pore size 1 month after treatment. Sebum and pore size on the side treated with BTX‐A continue to improve after 4 months of treatment [17]. Compared with the above studies, the present study indicated that the combination of BTX‐A and HA improved pores and texture. Patient satisfaction continued to improve significantly for 4 months. The effect of combining BTX‐A and HA was better than that of using BTX‐A alone because HA accumulated the effect of BTX‐A.

Sodium hyaluronate compound for injection contains sodium hyaluronate, proline, glycine, alanine, L‐carnosine, and vitamin B2. HA effectively moisturizes and hydrates the dermis; proline, glycine, and alanine can provide raw materials for fibroblasts to synthesize collagen and assist in improving pore size; L‐carnosine, a naturally water‐soluble dipeptide composed of β alanine and L histidine, can delay cell aging, maintain cell homeostasis, promote wound healing, and resist oxidation [18, 19]. Intradermal injection of low‐molecular‐weight HA can significantly improve skin texture, reduce pore size, and enhance skin brightness; however, it achieves only a 40.03% improvement rate [20]. A study showed that the combination of microfocused ultrasound and HA filler can be used for treating facial pore enlargement. Both of these techniques can effectively reduce the enlargement of facial pores and enhance patient satisfaction [21].

The combination of BTX‐A and HA achieves satisfactory results in the application around the eyes, chin, and corners of the mouth [22, 23, 24, 25]. BTX‐A and HA fillers improve small mouth and oral dysfunction in scleroderma [26]. A large multicenter, global retrospective study showed that patients who received a combination of HA and neuromodulators are more likely to retain the effects for many years than those who received both treatments separately; this finding suggests that combination therapy has a significant advantage in the duration of effectiveness retention [27]. We have reason to believe that BTX‐A and HA complement each other, and their combination is more effective than a single application. Our combined BTX‐A and HA compound injection therapy improved facial pore enlargement, and significant effects were observed 1 month after treatment. Despite a decrease in efficacy, the combination therapy still had significant effects on the conditions of skin pores, texture, and porphyrins compared with those at baseline, further confirming the clinical value of the combination therapy.

The combination of BTX‐A and HA in this study significantly improved severe facial pore enlargement. It is an effective and safe method for treating pore enlargement.

## Author Contributions

R.Y., X.M. and C.L. performed the research. X.M., R.Y. and M.Z. designed the research study. Y.B. and Y.Y. contributed essential reagents or tools. S.C. and J.L. analyzed the data. R.Y., S.C., X.M., and J.L. wrote the paper.

## Conflicts of Interest

The authors declare no conflicts of interest.

## Data Availability

Data sharing is not applicable to this article as no new data were created or analyzed in this study.
